# Detection of Extended-Spectrum β-Lactamase (ESBL) *E. coli* at Different Processing Stages in Three Broiler Abattoirs

**DOI:** 10.3390/microorganisms11102541

**Published:** 2023-10-12

**Authors:** Nina Langkabel, Janine Burgard, Sabrina Freter, Reinhard Fries, Diana Meemken, Lüppo Ellerbroek

**Affiliations:** 1 Working Group Meat Hygiene, Institute of Food Safety and Food Hygiene, School of Veterinary Medicine, Freie Universität Berlin, 14163 Berlin, Germany; 2 Veterinary Centre for Resistance Research, Freie Universität Berlin, 14163 Berlin, Germany; 3 Federal Ministry of Food and Agriculture, 10117 Berlin, Germany

**Keywords:** poultry, abattoir, extended-spectrum β-lactamase (ESBL)-producing *Escherichia coli*, food safety

## Abstract

The European Food Safety Authority (EFSA) identified extended-spectrum β-lactamase/AmpC β-lactamase (ESBL/AmpC)-producing *E. coli* as one of the main priority hazards for poultry. Different studies detected ESBL-producing *E. coli* at broiler fattening farms and in abattoirs, concluding that poultry meat is a potential source of human infection. Broiler breast skin samples taken in three abattoirs with different scalding techniques were examined for ESBL-producing *Escherichia* (*E*.) *coli* and their phylogenetic groups. A total of 307 ESBL-producing *E. coli* isolates were found, and the abattoir with conventional immersion scalding with thermal treatment of the water had the lowest incidence. Phylogroups D/E and B1 were mostly detected, while phylogroups C, D, and E were not detected. Phylogroup B2 was detected in low proportions. The phylogroups B2 and D are important as they have been associated with urinary tract infections in humans, but were only detected in low proportions at different processing stages in this study. Since the risk for the consumer of being infected via chicken meat with ESBL-producing *E. coli* and *E. coli* of highly pathogenic phylogroups cannot be excluded, good kitchen hygiene is of great importance.

## 1. Introduction

*Escherichia* (*E*.) *coli* is a member of the *Enterobacteriaceae* family and lives commensally in the gastrointestinal tract of humans and animals [1,2,3]. These bacteria can spread via fecal shedding to the environment or during meat production onto the processed food. In the framework of meat production, ensuring good slaughter hygiene and avoiding fecal contamination is of great importance [4,5,6]. As a result, the surveillance of *Enterobacteriaceae* counts on carcasses is regulated for different animal species in the European Union (EU) [7]. In this context, *E. coli* can act as an indicator for fecal contamination of carcasses and food. Thus, it was suggested that testing for *E. coli* should be performed in regard to process hygiene controls in broiler abattoirs [8,9,10]. Besides being commensals, some *E. coli* strains carry virulence factors and can cause severe diseases in humans [1]. As described by Clermont et al. (2000 and 2013) [11,12], classification of *E. coli* strains via their phylogenetic groups is possible with a polymerase chain reaction (PCR). Currently, the seven groups (A, B1, B2, C, D, E, and F), which are associated with *E. coli* and the *Escherichia* clades (I, III, IV, and V) originating from *E. coli*, are described as phylogenetic groups [11,12]. Phylogroups A and B1 are found mainly in commensal *E. coli* strains, whereas phylogroups B2 and D are associated with severe infections in humans [13,14]. Additionally, resistance against antimicrobials is of importance, since extended-spectrum β-lactamase/AmpC β-lactamase (ESBL/AmpC)-producing *E. coli* can be found in humans, food producing animals, and food products, and were identified as one of the main hazards to be covered in meat inspection of poultry [8,15,16,17]. Depending on the genes present, different ESBL types are distinguished, with TEM, SHV, and CTX-M being the most frequently reported representatives [15,17,18,19,20].

Poultry meat is the most commonly consumed meat worldwide [21]. Today’s slaughtering and processing of broilers for food production is a highly automated process with high hygienic standards. Despite that, foodborne infections associated with poultry meat consumption are frequently reported, mainly campylobacteriosis and salmonellosis [16]. Each processing step can have an influence on the bacterial load that broilers carry on their feathers when entering the abattoir [22,23,24]. While some process steps reduce the bacterial load of carcasses, others can lead to an increase [5,22,23,24,25,26,27]. One important processing stage, which can be seen as a combination stage, is the steps of scalding and plucking. Scalding and plucking are necessary to remove the feathers from the carcasses, which is of great importance because feces adhering to skin and feathers may lead to (cross-)contamination of the carcasses and meat during production [28]. Both processing steps were identified as having an influence on the bacterial load, but studies are contradictory, meaning that a decrease but also an increase was seen, depending on the abattoir investigated [5,23,29,30,31]. Despite all poultry line automation and current technical solutions, it remains impossible to produce poultry meat without bacterial contamination. Besides *Campylobacter* and *Salmonella*, the European Food Safety Authority (EFSA) identified ESBL/AmpC-producing *E. coli* as one of the main priority hazards for poultry meat [8], and the occurrence in broiler meat in the EU remained at high levels in 2020 [16]. However, decreasing trends of ESBL/AmpC-producing *E. coli* in broilers and turkeys were reported [32] in recent years. This is supported by different studies that detected ESBL-producing *E. coli* at broiler fattening farms, in abattoirs, and in retail meat, concluding that poultry meat is a potential source of human infection with ESBL/AmpC-producing *Enterobacteriaceae* [23,26,33,34,35,36,37,38,39,40,41]. Since each flock has its own bacterial spectrum and load, and each abattoir has to be seen as unique, individual hygiene control programs to reduce the bacterial loads of the carcasses through the production process in place need to be applied [8,26,42].

We conducted a study at three commercial broiler abattoirs that used different scalding techniques to investigate the occurrence of ESBL-producing *E. coli* on broiler breast skin samples at different processing steps from the beginning to end of the slaughter line.

## 2. Materials and Methods

### 2.1. Sampling

Between May 2016 and February 2018, breast skin samples of broilers were taken at three commercial broiler abattoirs in Germany and in The Netherlands, with slaughter capacities between 10,000 and 13,500 birds per hour. The slaughter lines were constructed in similar ways and operated with scalding temperatures between 51 °C and 55 °C. The installed scalding technique differed in the three abattoirs. Abattoir 1 operated a conventional immersion scalder with two scalding tanks. In Abattoir 2, an immersion scalder with three scalding tanks, where the scalding water was reused and underwent a thermal treatment before being reintroduced in the third scalding tank, was installed. The scalding principle of the immersion scalders is based on convection, and the carcasses are pulled through the scalding water. The so-called AeroScalder^®^ (Marel, Boxmeer, The Netherlands) [43] in Abattoir 3 uses hot, humid, saturated air as the scalding medium. The scalding principle of the AeroScalder^®^ is based on condensation, which means that the hot air that is blown between the feathers condenses at the colder surface of the carcasses. The temperature at the carcass surface is similar to the temperatures operating with low scalding temperatures, even though the hot, humid, saturated air is warmed to 57 °C.

Per abattoir, 320 breast skin samples without feathers from carcasses out of 48 flocks were taken at the following 5 sampling positions: (a) before scalding, (b) after scalding, (c) after plucking, (d) directly before chilling, and (e) after chilling. At each sampling position, one abattoir employee removed four carcasses, chosen randomly, successively from the slaughter line and held them head-down for sampling. For breast skin excision, two imaginary lines were located across the carcass, one between the joints where the wings join the body and another below the sternum approximately at the level of a line between the hip joints. On each side of the carcass, the ends of these two lines were then imagined as being connected with each other vertically, along the carcass length. For sampling, sterile stainless-steel forceps, and single-use scalpels (B. Braun Melsungen AG, Melsungen, Germany) were used. The breast skin was lifted slightly with the forceps, and incisions were made carefully along the imaginary lines to aseptically remove all the breast skin on the carcass. Each breast skin sample was placed in a sterile blender bag with lateral filter for volumes up to 400 mL (VWR International GmbH, Darmstadt, Germany) and cooled immediately to 4 °C. All samples were transported at 4 °C to the laboratory at Freie Universität Berlin, Germany, and were stored in a refrigerator that maintained a temperature of around 4 °C until the next morning, when the laboratory work started.

### 2.2. Bacteriological Examination and Statistical Analysis

Each breast skin sample was weighed before analysis and was blended in a 1:9 ratio with Luria Bertani broth (LB; Carl Roth GmbH & Co., KG, Karlsruhe, Germany) in the blender bag that had been used for sample transportation. Dilution series were created, and 0.05 mL from each dilution were dropped in duplicate on MacConkey No. 3 agar plates (Thermo Fisher Diagnostics GmbH (former Oxoid Deutschland GmbH), Wesel, Germany) + 1 μg/mL cefotaxime (AppliChem, Darmstadt, Germany) and streaked out with the pipette tip. In parallel, the remainder of the blended breast skin sample and LB was used for enrichment via incubation at 37 °C overnight and the next day, streaking it out with a loop on MacConkey No. 3 agar plates (Thermo Fisher Diagnostics GmbH (former Oxoid Deutschland GmbH), Wesel, Germany) + 1 μg/mL cefotaxime (AppliChem, Darmstadt, Germany).

Microbial analyses of ESBL-producing *E. coli* included an isolation step on MacConkey agar No. 3 + cefotaxime, matrix-assisted laser desorption/ionization time-of-flight mass spectrometry (MALDI-TOF MS) examination, disc diffusion testing, PCR for ESBL genes (*bla*_CTX-M_, *bla*_SHV_, and *bla*_TEM_), and gene sequencing. All ESBL-producing *E. coli* isolates identified using these described methods underwent an additional PCR test for identification of the phylogenetic groups of *E. coli*, following Clermont et al. (2013) [12].

As the first step, colony morphology on MacConkey No. 3 agar (Thermo Fisher Diagnostics GmbH (former Oxoid Deutschland GmbH), Wesel, Germany) + 1 μg/mL cefotaxime (AppliChem, Darmstadt, Germany) was described, and suspected ESBL/AmpC *E. coli* (pink to reddish) colonies were subcultured until pure colonies were obtained.

These pure, suspected ESBL/AmpC *E. coli* colonies were used for further identification and confirmation with MALDI-TOF MS (MALDI Microflex LT and Biotyper database, Bruker Daltonics, Bremen, Germany). Per each suspected ESBL/AmpC *E. coli* isolate, two fields on the MALDI-TOF MS target plate were covered with a small amount of colony material and overlayed with 1 μL matrix solution. For preparing 1 mL of the matrix solution, 500 µL acetonitrile (VWR International GmbH, Darmstadt, Germany), 475 µL distilled water (Aqua dest.; VWR International GmbH, Darmstadt, Germany), and 25 µL trifluoroacetic acid (Sigma-Aldrich, Chemie GmbH, Taufkirchen, Germany) were mixed for 2 min before 20 mg of α-cyano-4-hydroxycinnamic acid was added and the solution was mixed again for 3 min. After centrifugation, the supernatant was used as the matrix solution.

For isolates that were confirmed as *E. coli* with MALDI-TOF MS, antimicrobial susceptibility testing with disc diffusion tests for ESBL was performed according to the CLSI standards [44]. A suspension in 3 mL LB-broth (Carl Roth GmbH & Co., KG, Karlsruhe, Germany) was created per isolate and incubated at 37 °C for 6 to 24 h. From each culture, a suspension equivalent to McFarland standard 0.5 was prepared using a densitometer (Grant Bio DEN-1 McFarland Tube Densitometer, Grant Instruments, Cambridge, UK). The test discs for agar diffusion tests can be found in Table 1.

Additionally, the isolates were tested for the most common ESBL genes, *bla*_CTX-M_, *bla*_SHV_, and *bla*_TEM_, and additionally *bla*_CMY-2_, with a real-time PCR protocol [45]. For DNA extraction, isolates were incubated overnight at 37 °C in 3 mL LB-broth (Carl Roth GmbH & Co., KG, Karlsruhe, Germany). From each overnight culture, 1 mL was centrifuged (14,000 rpm, 4 min), and after discarding the supernatant, the resulting pellet was washed in 250 µL 0.1% TE buffer (VWR International GmbH, Darmstadt, Germany). After centrifugation of this solution (14,000 rpm, 4 min) and discarding of the supernatant, the pellet was resuspended in 250 µL 5% Chelex^®^ 100 sodium form solution (Sigma–Aldrich Chemie GmbH, Taufkirchen, Germany) and incubated at 56 °C (shaken at 700 rpm, 1 h), followed by incubation at 95 °C (shaken at 700 rpm, 15 min), and a final centrifugation step (14,000 rpm, 10 min). The resulting DNA extract was stored at 7 °C before analysis. The real-time PCR was performed using the Bio-Rad Real-Time PCR Detection System CFX96 (Bio-Rad Laboratories GmbH, Feldkirchen, Germany). The primers and probes as well as the PCR conditions followed the protocol set by Roschanki et al. (2014) [45]. The PCR-cycles used [45] were as follows:

- One cycle: denaturation        15 min, 95 °C
- Thirty cycles:
  o Denaturation                       15 s, 95 °C
  o Binding                                15 s, 50 °C
  o Elongation                           20 s, 70 °C

The real-time PCR results were evaluated using Bio-Rad CFX-Manager^TM^ 3.1 software (Bio-Rad Laboratories GmbH, Feldkirchen, Germany). All isolates that were CMY-positive were not used for further analyses because they were considered to be AmpC β-lactamase-producing *E. coli*. All isolates encoding for CTX were considered as ESBL-producing *E. coli*.

From isolates in which only *bla*_TEM_ or *bla*_SHV_ was found, DNA was extracted with GeneJet PCR Purification Kit (Thermo Fisher Scientific Baltics UAB, Vilnius, Lithuania) following the manufacturer’s instructions. DNA purity and concentration was measured with NanoDrop^TM^ 2000 (Thermo Fisher, (Thermo Fisher Scientific, Wilmington, MA, USA). After the assessment, pure DNA (200 ng/µL to 100 ng/µL) with primer sequences for *bla*_TEM_ or *bla*_SHV_, as described by Projahn et al. (2017) [46], was sent for gene sequencing to Eurofins Genomics (Eurofins GATC Biotech GmbH, Konstanz, Germany).

In the last step, phylogenetic groups for all ESBL-producing *E. coli* isolates were identified using the PCR protocol according to Clermont et al. (2013) [12].

For statistical analysis, this study was planned to utilize descriptive analysis of resistance encoding genes at three different abattoirs. The descriptive statistical analyses were carried out using IBM^®^ SPSS^®^ version 28.0.1.0 for Windows and figures were built with Microsoft^®^ Excel^®^ LTSC Professional Plus 2021 version 2108. For testing the association between phylogenetic groups and resistance encoding genes, the Fisher exact test was performed; *p*-values < 0.05 were regarded as statistically significant.

## 3. Results

In total, 960 breast skin samples were analyzed, and after the stepwise analysis for ESBL-producing *E. coli*, 307 isolates were finally confirmed. From some samples, more than one isolate was obtained. For further analysis, only the ESBL-producing *E. coli* isolates obtained were used.

**Figure 1:**
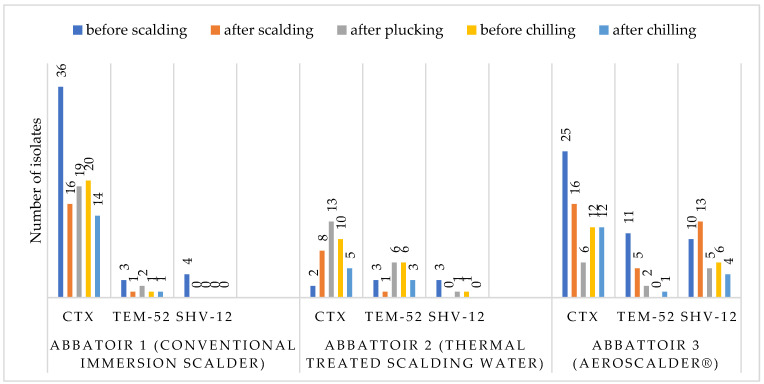
Extended-spectrum β-lactamase (ESBL)-producing *E. coli* isolates from breast skin samples from Abattoir 1 (*n* = 117; using a conventional immersion scalder), Abattoir 2 (*n* = 62; using immersion scalding with thermal treatment of the scalding water), and Abattoir 3 (*n* = 128, operating the AeroScalder^®^), and number of isolates encoding ESBL genes detected at the sampling positions.

The frequency of ESBL-producing *E. coli* detection varied between the three abattoirs and between the different sampling positions. Abattoir 3 with the AeroScalder^®^ showed the highest frequency with 128/307 (41.7%) isolates, followed by Abattoir 1 that used an immersion scalder with 117/307 (38.1%) isolates, and then Abattoir 2 that used an immersion scalder with thermal treatment of the water with the lowest frequency with 62/307 (20.1%) isolates. ESBL-producing *E. coli* were found at all sampling positions, with higher incidences at the beginning of the slaughter process except for Abattoir 2, where an increase after plucking was seen (Figure 1).

The frequency of the encoding genes in total was the highest for *bla*_CTX-M_ (69.7%, 214/307), followed by *bla*_TEM-52_ (14.9%, 46/307), and *bla*_SHV-12_ (15.3%, 47/307). In Abattoir 1, *bla*_CTX-M_ was the most frequently found encoding gene in all sampling positions. *Bla*_SHV-12_ was found in four isolates before scalding, two of them from birds out of one flock. In Abattoir 3, mostly high frequencies of *bla*_CTX-M_ and *bla*_SHV-12_ were found at all sampling positions; however, *bla*_TEM-52_ was not found before chilling (Figure 1).

The antimicrobial susceptibility test using agar diffusion was performed on all 307 isolates; however, results were only obtained for 302 isolates because 5 isolates were non-culturable despite our standard laboratory culture and storage practices. Of these 302 isolates, 6 isolates were susceptible to CTX but harbored TEM-52 (1/6) and/or SHV-12 (5/6) encoding genes. These isolates were obtained from all abattoirs, with three isolates from one flock at Abattoir 2. In total, 28 isolates were resistant to 2 of the tested antibiotics (FOX-CTX: 1/28 (3.6%); CAZ-CTX: 27/28 (96.4%)). The isolates that were resistant to CAZ and CTX were found on different sampling days at all three abattoirs (11 at Abattoir 1, 1 at Abattoir 2, and 15 at Abattoir 3). At Abattoir 1, three isolates were found on an early sampling day and belonged to the same flock, and another seven isolates from a later sampling day were also from just one flock. At Abattoir 3, two isolates from one flock were found on one of the sampling days. On a later sampling day, eleven isolates were found, of which six belonged to the first flock sampled that day and five to the second flock sampled (see Supplementary Materials. Overall, four isolates were resistant to three of the tested antibiotics (FOX-CAZ-CTX: 3/4 (75%); CAZ-CTX-MRP: 1/4 (25%)). Two of the three isolates that were resistant to FOX, CAZ, and CTX were from the same flock, sampled at Abattoir 2 after chilling. The other two isolates with threefold antibiotic resistance were found before scalding at Abattoir 1 and Abattoir 3, respectively (see Supplementary Materials).

Various *E. coli* phylogroups were found at the three abattoirs. No isolate belonged to phylogroups C, D, E, or E/*E*. clades. At Abattoir 1 (using conventional immersion scalding), predominant phylogroups were phylogroups D/E (76/117) and A/C (28/117). In total, just two isolates at the sampling position before scalding and one isolate after chilling belonged to phylogroup B2 (Table 2). At Abattoir 2 (using conventional immersion scalding with thermal treatment of the scalding water), most isolates belonged to *E. coli* phylogroup B1 (38/63), followed by phylogroup B2 (14/63). The isolates from phylogroup B2 were found after scalding (1), after plucking (8), before chilling (4), and after chilling (1). In addition to the abovementioned undetected phylogroups, phylogroup F was not found at Abattoir 2 (Table 2). The most frequently found phylogroup at Abattoir 3 (using the AeroScalder^®^) was phylogroup D/E (61/128), followed by phylogroups B1 (36/128) and A/C (25/128). Three isolates belonged to phylogroup B2; two were found before scalding and one after plucking. After chilling, phylogroup B2 was not found. Phylogroups A and F were not found at Abattoir 3 in addition to the abovementioned undetected phylogroups (Table 2).

The resistance profiles of the 20 phylogroup B2 isolates are presented in Table 3. All isolates were meropenem resistant. One isolate at Abattoir 1 before scalding was one of the CTX susceptible isolates. One of the 20 isolates was resistant to two antibiotics (CAZ-CTX) and was found after plucking at Abattoir 2. At Abattoir 3, one of the phylogroup B2 isolates after chilling was resistant to three of the tested antibiotics (FOX-CAZ-CTX). All other isolates were resistant to cefotaxime (CTX) but susceptible to or intermediate to the other three antibiotics tested (Table 3).

Statistical comparison of the associations between phylogroups and resistance encoding genes showed that phylogroup B1 and *bla*_TEM-52_ (*p* < 0.001) and that phylogroup A/C and *bla*_SHV-12_ (*p* < 0.005) had a more frequent statistically significant association with each other. There was no statistically significant association between *bla*_CTX-M_ and any of the phylogroups found. Phylogroup B2 was also not significantly associated with any of the resistance encoding genes.

## 4. Discussion

In total, 307 ESBL-producing *E. coli* isolates were found in 960 broiler breast skin samples taken in the 3 studied abattoirs. As in previous investigations [15,47,48], *bla*_CTX-M_ was the main gene encoding for antimicrobial resistance found in this study. The occurrence of the antimicrobial resistance genes differed along the slaughter line among the three abattoirs. This finding supports the conclusions of previous studies—that each abattoir is unique regarding the distribution and occurrence of bacteria, and that the microbial loads are influenced by the flock and the abattoir itself [8,27,42,49].

Comparing the three abattoirs, it can be seen that in Abattoir 2, using a conventional immersion scalding tank where the scalding water underwent a thermal treatment before being reintroduced to the scalding tank, the lowest numbers of ESBL-producing *E. coli* isolates were found, both overall and after scalding. In Abattoir 3, operating the AeroScalder^®^, which uses hot, humid, saturated air as the scalding medium, the highest numbers of ESBL-producing *E. coli* were found after scalding. This finding could be explained by the fact that a washing effect, as in the other abattoirs using immersion scalders, was missing. However, after plucking, the numbers of ESBL-producing *E. coli* were reduced to a lower incidence than was found in Abattoirs 1 and 2. At the end of the slaughter line before chilling, similar numbers of ESBL-producing *E. coli* isolates were found in all three abattoirs. After chilling in Abattoir 2, the lowest number of ESBL-producing *E. coli* was found (among the three abattoirs). We could not identify a single production step as having the main influence on reducing ESBL-producing *E. coli* in the abattoirs studied. Since ESBL-producing *E. coli* were found after chilling in all three abattoirs, a risk of transmission to the consumer via chicken products on the market must be assumed. Other studies showed frequent findings of ESBL-producing *E. coli* in broiler abattoirs, including findings in abattoir workers, and proved that this risk exists [16,26,50,51,52].

In Abattoir 2, compared with the other abattoirs, the numbers of ESBL-producing *E. coli* were the lowest, specifically from the beginning of production and onwards. Therefore, it seems likely that the status of the incoming flock is of great importance, as incoming flocks carrying ESBL-producing *E. coli* could lead to these microorganisms being distributed through the abattoir. Similar findings were reported from different studies investigating the occurrence of ESBL-producing *E. coli* in German broiler fattening farms and the surrounding environment [35,36,53,54].

A great variety of phylogroups was seen; however, of the phylogroups known to be associated with severe human diseases, such as urinary tract infections or meningitis [55,56,57], only phylogroup B2 was found, as well as in low numbers compared to the phylogroups known to be linked to apathogenic *E. coli* strains [13]. Interestingly, the highly virulent phylogroup B2 was found in the highest numbers (especially after plucking), in Abattoir 2, in which the lowest numbers of ESBL-producing *E. coli* were detected. Thus, it can be assumed that in that unique case, cross-contamination from the plucking fingers was apparent, meaning that the flocks slaughtered before the flocks sampled harbored *E. coli* phylogroup B2, which were then transferred to the carcasses sampled during plucking. Persistence of *E. coli* in abattoirs [52] can be a source of contamination during processing.

Regarding the phenotypic resistance profiles, it can be seen that different resistance profiles were found on different sampling days and at different sampling positions. Since we sometimes found the same resistance profiles on the same sampling day, and often in samples belonging to one flock, it is possible that the birds themselves were carriers of *E. coli* with the respective resistance profiles or that transmission occurred between the birds during slaughter. The detection of *E. coli* with the same resistance profile in two consecutive flocks sampled on one day could also be explained by transmission from the first to the latter slaughtered flock. Since we did not sample the plucking fingers before our sampling, we can only assume this possibility, but Pacholewicz et al. (2015) [26] showed decreases in ESBL-producing *E. coli* after defeathering. In contrast, other authors reported increases of microbial loads after plucking [58,59,60]; therefore, the risk of cross-contamination during processing must be considered. However, since we did not find *E. coli* with the same resistance profile in the following sampling days at sampling positions after scalding, it seems that the incoming birds were more likely a source of the ESBL-producing *E. coli* rather than an accumulation of these bacteria in the abattoir. Therefore, it can be assumed that the cleaning and disinfection procedures in the abattoirs were a control method against the accumulation of EBSL-producing *E. coli*. Nonetheless, it must be taken into consideration that some of our examined breast skin samples harbored ESBL-producing *E. coli*; thus, the hygiene during the cutting of poultry carcasses is important because, during that process, bacteria can transfer from the outer parts of the carcasses to the meat and processed products. Overall, the pathogenic phylogroup B2 was only found twice after chilling in different abattoirs; however, in one case, it showed a threefold antibiotic resistance, as shown before in poultry meat [61]. Therefore, it seems that broilers probably have a low prevalence of phylogroup B2, as was also shown in a study by Reich et al. (2013) [33]. Nevertheless, highly virulent, and highly resistant ESBL-producing *E. coli* can still occur on the carcasses after chilling (like in this study). On the other hand, phylogroups representing commensal *E. coli* were mainly found by us, and it can be concluded that the risk of severe infections seems to be low. However, the fact that all isolates examined were ESBL-producing *E. coli* with various resistance profiles must be considered in the context of chicken meat safety, as it shows that transmission to humans via this food is possible, even if the risk appears to be low from our point of view.

## 5. Conclusions

In the three abattoirs investigated, we determined that the different scalding techniques in combination with the different incoming flocks lead to the reductions in different bacteria. ESBL-producing *E. coli* and human pathogenic *E. coli* were found at different processing steps along the slaughter lines and were able to contaminate broiler carcasses after scalding and even after chilling.

The risk of transmission, via chicken meat consumption, of antimicrobial resistant *E. coli* with phylogenic group profiles that can cause serious illness in humans cannot be excluded. Therefore, good hygiene during cutting and adherence to good kitchen hygiene guidelines, which are mainly used to prevent *Campylobacter* and *Salmonella* infections via raw chicken meat, should also be considered important for preventing infections with ESBL-producing *E. coli* via chicken meat. In addition, further research should focus on the individual microbiota of slaughterhouse operations and how the component bacteria are affected by processing, management, and daily cleaning and disinfection procedures.

## Figures and Tables

**Table 1:** Test discs used for agar diffusion test.

Antimicrobial	Abbreviation	Concentration	Manufacturer
Cefotaxime	CTX	30 µg	Thermo Fisher Diagnostics GmbH (former Oxoid Deutschland GmbH), Wesel, Germany
Cefoxitin	FOX	30 µg	
Ceftazidime	CAZ	30 µg	
Meropenem	MRP	10 µg	
Cefotaxime and clavulanic acid	CTL	30/10 µg	Bestbion, Hürth, Germany

**Table 2:** Detected phylogroups of extended-spectrum β-lactamase (ESBL)-producing *E. coli* isolates at Abattoir 1 (using conventional immersion scalding; *n* = 117), Abattoir 2 (using conventional immersion scalding with thermal treatment of the scalding water; *n* = 62), and Abattoir 3 (using the AeroScalder^®^; *n* = 128) per sampling position.

	Phylogroup	Sampling Position						
		Before Scalding	After Scalding	After Plucking	Before Chilling	After Chilling	Total	Total Overall
Abattoir 1 (conventional immersion scalder)	A	0	0	0	1	0	1	117
	A/C	7	3	7	5	6	28	
	B1	5	2	0	1	0	8	
	B2	2	0	0	0	1	3	
	D/E	27	12	15	14	8	76	
	F	1	0	0	0	0	1	
	not assigned to any group	0	0	0	0	0	0	
Abattoir 2 (conventional immersion scalder with thermal treatment of the scalding water)	A	1	0	0	0	0	1	62
	A/C	0	0	4	2	0	6	
	B1	4	8	8	11	7	38	
	B2	0	1	8	4	1	14	
	D/E	3	0	0	0	0	3	
	F	0	0	0	0	0	0	
	not assigned to any group	0	0	0	0	0	0	
Abattoir 3 (AeroScalder^®^)	A	0	0	0	0	0	0	128
	A/C	9	8	2	3	3	25	
	B1	12	10	4	4	6	36	
	B2	2	0	1	0	0	3	
	D/E	22	14	6	11	8	61	
	F	0	0	0	0	0	0	
	not assigned to any group	1	2	0	0	0	3	

**Table 3:** Resistance profiles of 20 *E. coli* phylogroup B2 isolates per abattoir and sampling position.

Lab-ID	Sampling Day	Abattoir	Sampling Position	FOX	CAZ	CTX	MRP	CTL-CTX Diameter (mm)
1215	5	1	before scalding	S	I	S	S	8
1216	5		before scalding	S	I	R	S	10
380	10		after chilling	S	S	R	S	14
984	4	2	after scalding	S	S	R	S	17
988	4		after plucking	S	I	R	S	15
990	4		after plucking	S	S	R	S	11
992	4		after plucking	S	S	R	S	12
994	4		after plucking	S	R	R	S	10
1020	4		after plucking	S	S	R	S	9
1022	4		after plucking	S	S	R	S	9
1024	4		after plucking	S	I	R	S	12
1026	4		after plucking	S	I	R	S	16
996	4		before chilling	S	S	R	S	12
998	4		before chilling	S	S	R	S	15
1000	4		before chilling	S	n.d.	R	S	16
1030	4		before chilling	S	S	R	S	16
63	8		after chilling	R	R	R	S	6
702	2	3	before scalding	S	I	R	S	15
703-1	2		before scalding	S	S	R	S	14
689	2		after plucking	S	I	R	S	14

ID—laboratory identification number; FOX—cefoxitin 30 µg; CAZ—ceftazidime 30 µg; CTX—cefotaxime 30 µg; CTL—cefotaxime and clavulanic acid 30/10 µg; S—susceptible; I—intermediate; R—resistant; n.d.—no data.

## Data Availability

The data presented in this study are available upon request from the corresponding author. Data that can identify the abattoirs are not publicly available due to privacy concerns.

## Supplementary Materials

The following supporting information can be downloaded at: https://www.mdpi.com/article/10.3390/microorganisms11102541/s1. Table S1: Results ESBL positive isolates.
